# Incipient Plasticity of Si and GaAs: Review and Perspectives

**DOI:** 10.3390/ma18174011

**Published:** 2025-08-27

**Authors:** Dariusz Chrobak

**Affiliations:** Institute of Materials Engineering, University of Silesia in Katowice, ul. 75 Pułku Piechoty 1A, 41-500 Chorzów, Poland; dariusz.chrobak@us.edu.pl

**Keywords:** incipient plasticity, semiconductors, silicon, gallium arsenide, nanoindentation, classical molecular dynamics

## Abstract

Despite the remarkable developments in advanced materials, silicon and gallium arsenide remain among the leading semiconductors of our time. Nanomechanical studies of these semiconductor crystals, including nanoindentation-induced structural phase transformations and dislocation generation, remain important for science and technology. Of particular interest are studies on the onset of plasticity. What phenomenon initiates plastic deformation in Si and GaAs during nanoindentation? Through complex experiments and computer simulations, significant progress has been made in answering this question over the past twenty years. Indeed, equipping nanoindentation systems with the ability to record Raman spectra and exploring new interatomic interaction models for classical molecular dynamics have opened up new avenues for studying the non-trivial interplay between structural phase transformations and dislocation activity in semiconductor crystals. The diversity of high-pressure phases, especially silicon, and the largely unexplored sequences of transformations between them continue to inspire new scientific challenges. This article reviews selected works introducing the reader to the fascinating and still open topic of nanoindentation-induced incipient plasticity in silicon and gallium arsenide.

## 1. Introduction

Progress in understanding the course of initial plasticity in semiconductors is related to the development of the nanoindentation method [1]. Sharp indenters (apex radius ∼100 nm) allow for studying the material’s mechanical response in nanovolumes free from linear defects. Basic nanoindentation experiments can be enriched with measurements of the electrical conductivity [2], the acoustic emission [3], and the Raman spectroscopy [4] Due to the small volume of the crystal subjected to stress during nanoindentation, using methods for studying structural changes occurring in investigated crystals is extremely difficult. Therefore, the outcomes of nanoindentation experiments are often supplemented with molecular dynamics modeling [5], which allows for interpreting research results at the atomic level.

In the vicinity of the contact between the indenter tip and the semiconductor surface, a stress distribution is created, which, in the case of studying the crystal free of dislocations, can cause their nucleation and induce a sequence of structural phase transformations. It is important to note that apart from its displacement axis, the spherical indenter does not favor any other crystallographic direction. The influence of dislocation nucleation and phase transformations on the onset and continuation of plastic deformation is not evident in the case of semiconductor crystals. While in metals such as Ag and Al, the A1 type structure is stable in the range of compressive stresses not exceeding 240 GPa [6] and 217 GPa [7], respectively. In contrast, several or dozens of GPa stresses are sufficient to initiate phase transformations in several semiconductors (e.g., Si, Ge, GaAs, GaP, InP, CdTe). This stress level is easily achieved during nanoindentation, generating shear stresses of a value sufficient to nucleate dislocations. Consequently, nanoindentation-induced plastic deformation of semiconductor crystals is a complex physical phenomenon combining dislocation activity and structural phase transformations. Progress in understanding this issue influences the miniaturization of electromechanical devices (sensors, actuators), in which the contact area of micro- or nano-elements is extremely small [8,9]. In such specific contact, forces acting on it can be a source of significant stresses leading to changes in the structure of contacting elements, which affects the functional properties of designed systems.

This review presents the current knowledge concerning the processes initiating plastic deformation in Si and GaAs semiconductor crystals, focusing on the competition between structural phase transformations and dislocation activity at the moment of the elastic–plastic transition.

An interesting study on nanoindentation problems of other semiconductor crystals (e.g., Ge, GaN, InP, CdZnTe, HgCdTe) can be found in the review by Sharma et al. [10].

## 2. Nanoindentation

The nanoindentation method allows for studying the mechanical response of a tested crystal subjected to the stress developed in a small volume right below the contact between the indenter and the crystal surface. Considering that the average distance between dislocations can be many times greater than the radius of the indenter tip, the nanoindentation method is suitable for studying the mechanical properties of samples with a structure similar to that of an ideal single crystal.

The nanoindentation results are presented as P(h) curves describing the relationship between the load *P* applied to the indenter and its displacement *h*. On this basis, it is possible to determine such material constants as the reduced Young’s modulus $E_{r}$ and the nanoindentation hardness *H*. The discrete nature of plastic deformation processes causes the appearance of characteristic singularities on the P(h) curve, which can result from both the development of the dislocation network and structural phase transformations.

### 2.1. Elements of the Contact Theory

**Figure 1:**
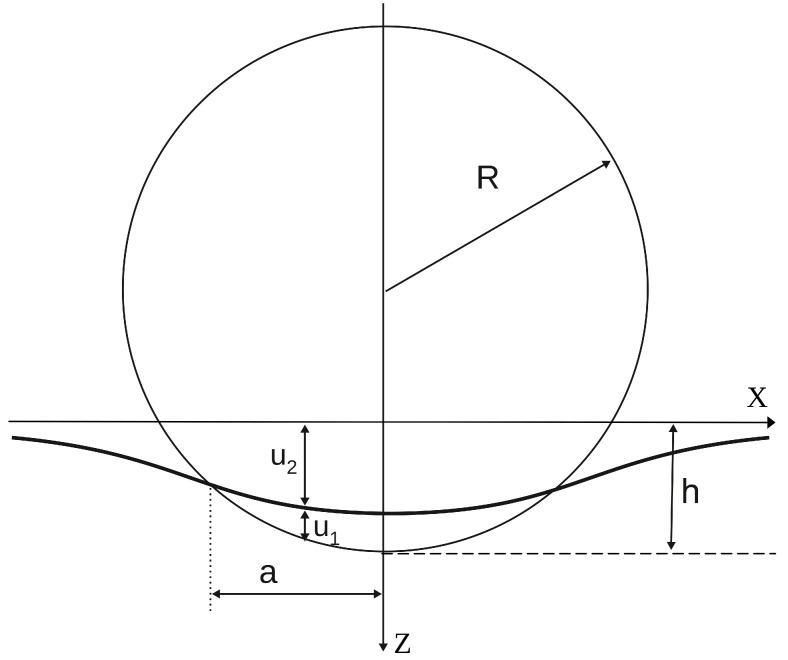
Indentation geometry with an elastic spherical indenter (1) of the radius *R*: *a*—the contact radius, *h*—the displacement of the indenter. The thick continuous line is a trace of the deformation of the half-space (z⩾0) boundary filled with an elastic medium (2).

The analysis of nanoindentation results is based on the Hertzian theory of elastic contact of two homogeneous and non-conforming bodies [11]. The assumption that the contact surface area is small enables the application of the linear theory of elasticity. Additionally, friction effects are neglected, which means that the only component perpendicular to the contact surface is “transmitted” from one body to the second. As the shape of the indeter tip is often approximated by a spherical surface, the case of contact of a sphere of radius *R* (Young’s modulus $E_{1}$, Poisson’s ratio $\nu_{1}$) with a half-space filled with the tested material ($E_{2}$, $\nu_{2}$ ) will be considered below (Figure 1).

The measure of the deformation of both contacting objects is the displacement of points lying on the contact surface ($u_{1}$ and $u_{2}$), which for small *h* satisfy the following equation:

(1) $$u_{1}+u_{2}=h-\frac{1}{2R}r^{2},$$

where $r=\sqrt{x^{2}+y^{2}}⩽a$.

**Figure 2:**
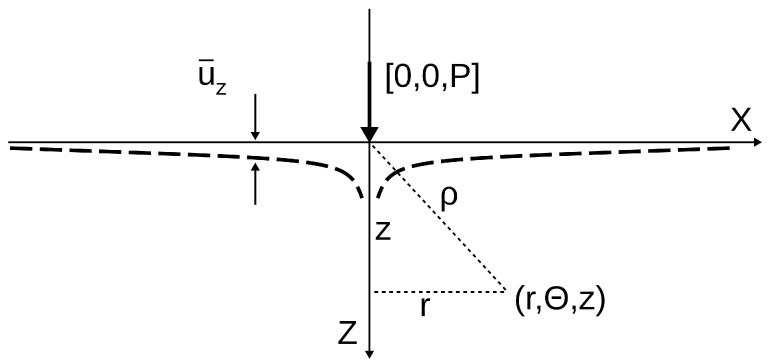
Deformation $u_{z}$ of a surface under the action of a point force [0,0,P] applied at the origin of the coordination system. The deformation asymptotically approaches zero as r→∞ and has a singularity at r=0.

In order to determine the relationship between the force acting on the indenter and its displacement, it is convenient to consider first the reaction of the elastic medium filling the half-space (z⩾0) to the action of a point force acting on its boundary [12] (Figure 2). Then, using the principle of superposition, the result can be generalized to a continuous distribution of point forces.

If the point force $P=P{\hat{e}}_{z}$ (Figure 2) is applied at the origin of the coordinate system, then the non-zero components of the stress tensor ($\sigma_{rr},\sigma_{\theta \theta },\sigma_{zz},\tau_{rz}$) and the displacement vector ($u=u_{r}{\hat{e}}_{r}+u_{\theta }{\hat{e}}_{\theta }+u_{z}{\hat{e}}_{z}$) are represented by the following Equations [11,12]:

(2) $$\begin{array}{ccc} \sigma_{rr} & = & \frac{P}{2\pi }\left\{\left(1-2\nu \right)\left[\frac{1}{r^{2}}-\frac{z}{r^{2}\rho }\right]-\frac{3r^{2}z}{\rho^{5}}\right\}\\ \sigma_{\theta \theta } & = & -\frac{P}{2\pi }\left(1-2\nu \right)\left\{-\frac{1}{r^{2}}-\frac{z}{r^{2}\rho }+\frac{z}{\rho^{3}}\right\}\\ \sigma_{zz} & = & -\frac{3P}{2\pi }\frac{z^{3}}{\rho^{5}}\\ \tau_{rz} & = & -\frac{3P}{2\pi }\frac{rz^{2}}{\rho^{5}}, \end{array}$$

(3) $$\begin{array}{ccc} u_{r} & = & \frac{P}{4\pi G}\left\{\frac{rz}{\rho^{3}}-\left(1-2\nu \right)\frac{\rho -z}{r\rho }\right\}\\ u_{z} & = & \frac{P}{4\pi G}\left\{\frac{z^{2}}{\rho^{3}}+\frac{2(1-\nu )}{\rho }\right\}, \end{array}$$

where $G=\frac{E}{2(1+\nu )}$ is the shear modulus. Consequently, the displacement of a point lying on the half-space boundary located at a distance *r* from the origin of the coordinate system is given by:

(4) $$\begin{array}{ccc} {\bar{u}}_{r} & = & -\frac{\left(1-2\nu \right)}{4\pi G}\frac{P}{r}\\ {\bar{u}}_{z} & = & \frac{\left(1-\nu \right)}{2\pi G}\frac{P}{r}. \end{array}$$

The above result can be generalized to the case of a continuous load distribution and a flat contact surface (shallow indentation). The displacement ${\bar{u}}_{z}$ at any point (x,y) of the contact surface is the sum of elementary displacements induced by the force acting on the elements $dx^{\prime }dy^{\prime }$ belonging to the contact surface *S*. Denoting the contact pressure distribution by $p_{c}\left(x^{\prime },y^{\prime }\right)$, one can write:

(5) $$\begin{array}{ccc} u_{1}\left(x,y\right) & = & \frac{\left(1-{\nu_{1}}^{2}\right)}{\pi E_{1}}\underset{S}{\;\int \int \;}\frac{p_{c}\left(x^{\prime },y^{\prime }\right)}{\sqrt{{\left(x-x^{\prime }\right)}^{2}+{\left(y-y^{\prime }\right)}^{2}}}dx^{\prime }dy^{\prime }\\ u_{2}\left(x,y\right) & = & \frac{\left(1-{\nu_{2}}^{2}\right)}{\pi E_{2}}\underset{S}{\;\int \int \;}\frac{p_{c}\left(x^{\prime },y^{\prime }\right)}{\sqrt{{\left(x-x^{\prime }\right)}^{2}+{\left(y-y^{\prime }\right)}^{2}}}dx^{\prime }dy^{\prime } \end{array}$$

where $\sqrt{x^{\prime 2}+y^{\prime 2}}\leq a$. After substituting to Equation (1), the relationship between the contact pressure distribution $p_{c}\left(x^{\prime },y^{\prime }\right)$ and the sphere displacement *h* takes the form:

(6) $$\underset{S}{\;\int \int \;}\frac{p_{c}\left(x^{\prime },y^{\prime }\right)}{\sqrt{{\left(x-x^{\prime }\right)}^{2}+{\left(y-y^{\prime }\right)}^{2}}}dx^{\prime }dy^{\prime }=\pi E_{r}\left\{h-\frac{1}{2R}\left(x^{2}+y^{2}\right)\right\},$$

where $1/E_{r}=\left(1-{\nu_{1}}^{2}\right)/E_{1}+\left(1-{\nu_{2}}^{2}\right)/E_{2}$ is the inverse of the reduced Young’s modulus.

The following integral attracts a special attention:

(7) $$\underset{\sqrt{x^{\prime 2}+y^{\prime 2}}\leq a}{\;\int \int \;}\sqrt{1-{\left(\frac{x^{\prime }}{a}\right)}^{2}-{\left(\frac{y^{\prime }}{a}\right)}^{2}}\frac{dx^{\prime }dy^{\prime }}{\sqrt{{\left(x-x^{\prime }\right)}^{2}+{\left(y-y^{\prime }\right)}^{2}}}=\frac{\pi^{2}}{4a}\left\{2a^{2}-\left(x^{2}+y^{2}\right)\right\},$$

When compared with Equation (6), it allows us to determine the form of the pressure distribution contact

(8) $$p_{c}\left(x,y\right)=p_{0}\sqrt{1-{\left(\frac{x}{a}\right)}^{2}-{\left(\frac{y}{a}\right)}^{2}}.$$

The constant $p_{0}$ is related to the load *P*:

(9) $$\begin{array}{ccc} P & = & p_{0}\underset{S}{\;\int \int \;}\sqrt{1-{\left(\frac{x}{a}\right)}^{2}-{\left(\frac{y}{a}\right)}^{2}}dxdy=p_{0}\frac{2\pi a^{2}}{3},\\ p_{0} & = & \frac{3P}{2\pi a^{2}}=\frac{3}{2}p_{m}, \end{array}$$

where $p_{m}=P/\left(\pi a^{2}\right)$ is the average pressure at the contact surface (contact pressure).

From Equations (6), (7) and using (9) one can obtain the following relationships:

(10) $$a^{2}=Rh$$

(11) $$P=\frac{4}{3}E_{r}\frac{a^{3}}{R}$$

which results in the famous Hertz equation for elastic deformation, relating the indenter load value *P* to its displacement *h*:

(12) $$P=\frac{4}{3}E_{r}\sqrt{R}h^{3/2}$$

Exact solutions to axisymmetric contact problems using a method of dimensionality reduction (MDR) are given in the book by Popov et al. [13].

**Figure 3:**
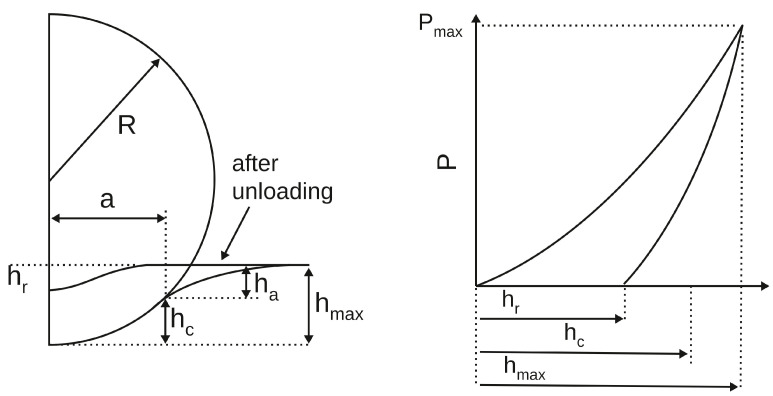
The Oliver–Pharr method. Illustration of the relationship between the maximum indentation depth $h_{max}$, the contact depth $h_{c}$, and the displacement of the surface at the contact perimeter $h_{a}$. $h_{r}$ is the depth of the residual indent impression.

Figure 3 schematically represents the elastic contact of a rigid sphere with a half-space filled with the tested medium. It allows us to write down the geometric relationship between the contact radius *a*, the indenter radius *R*, and the contact depth $h_{c}$: $a^{2}=2Rh_{c}$, for small $h_{c}$. Comparing this equation with (10), we get:

(13) $$h_{c}=\frac{1}{2}h$$

A special role in the theory of indentation is played by the stiffness coefficient *S*, which for the case of a spherical indenter takes the form:

(14) $$S=\frac{dP}{dh}=2E_{r}\sqrt{Rh}=\frac{2}{\sqrt{\pi }}E_{r}\sqrt{A}$$

where $A=\pi a^{2}=\pi 2Rh_{c}=\pi Rh$ is the contact area. Indeed, the Oliver–Pharr method [14] is based on an equation of a form similar to (14) and allows, by analyzing the unloaded part of the P(h) curve, to determine the reduced Young’s modulus $E_{r}$ also in the case when indentation is accompanied by plastic deformation. The method can be simply explained in the case of the spherical indentation and comes down to estimating the contact depth $h_{c}$ and then the contact area $A=2\pi Rh_{c}$. Assuming that the initial small segment of the unloading part of the curve P(h) reflects the elastic behavior of the material, one can imagine an equivalent elastic indentation passing through the points of that segment. Then, a short analysis based on Equations (12) and (13) leads to the following result:

(15) $$h_{c}=h_{max}-\frac{3}{4}\frac{P_{max}}{S_{max}}$$

where $S_{max}$ is calculated at the $\left(h_{max},P_{max}\right)$ point of the unloading curve.

Pharr et al. [15] used the results of Sneddon [16] and King [17] and showed that the stiffness coefficient given by formula (14) is valid for indenters with axial symmetry (solid of revolution). However, for indenters with triangular (Berkovich) and square (Vickers) cross-sections, a slight modification is necessary:

(16) $$S=\beta \frac{2}{\sqrt{\pi }}E_{r}\sqrt{A}$$

where β=1, β=1.034, β=1.012 for spherical, Berkovich, and Vickers indenters, respectively.

Also Equation (26) has to be modified [14] for non-spherical indenter geometries:

(17) $$h_{c}=h_{max}-\epsilon \frac{P_{max}}{S_{max}}$$

where ϵ=0.72, ϵ=0.75, and ϵ=1 for conical, spherical and Berkovich, and flat punch indenters, respectively.

The contact area *A* is not a simple function of the contact depth $h_{c}$, as for the spherical indentation it was. Within the frame of the Oliver–Pharr method, this dependence is expressed in the form of the following series [14]:

(18) $$A=F\left(h_{c}\right)=c_{0}h_{c}^{2}+\sum_{n=1}^{4}c_{n}h_{c}^{\frac{1}{2^{n-1}}}$$

where $c_{0}=24.5$ and the remaining parameters $c_{n}$ can be determined experimentally using an appropriate standard, for example, amorphous ${\mathrm{SiO}}_{2}$ (*fused silica*) with the reduced Young’s modulus $E_{r}=69.6$ GPa.

The scientific literature contains a number of papers utilizing the nanoindentation technique. In particular, the method’s excellent sensitivity was utilized in studies of the effect of pyrolysis temperature on the nature of bonds in glassy carbon by Jurkiewicz et al. [18]. A correlation was demonstrated between the $sp^{2}$-hybridized non-planar bond content and the indentation modulus. The maxima of these physical quantities corresponded. Looking for nanoindentation applications, one can point to work by Acosta-Silva et al. [19], which studied the photocatalytic properties of a composite system consisting of ${\mathrm{TiO}}_{2}$ layers on a ${\mathrm{SiO}}_{2}$ substrate. In this case, nanoindentation and nanoscratching could be used to investigate the mechanical stability of this multilayer system. In another study by Wu et al. [20], a titanium interlayer was used to strengthen the bond between the Cu matrix and the graphene network layer (GN). This resulted in the formation of ${\mathrm{Cu}}_{x}{\mathrm{Ti}}_{y}$ and TiC compounds in the interlayer, resulting in a significant increase in the strength and strain of the interfacial separation of the resulting composite. In this context, the idea of using nanoindentation to investigate the mechanical properties of the GN/Ti/Cu sandwich seems very interesting.

### 2.2. Indentation of the Anisotropic Medium

The importance of contact stiffness *S* for determining the reduced Young’s modulus of an isotropic medium was demonstrated above. The same applies to an anisotropic medium. A good starting point is the work of Vlassak and Nix published in 1993 [21]. Using the results by Willis [22] concerning the Boussinesq problem for an anisotropic half-space, the authors derived the following formula for the circular contact of the axisymmetric paraboloid indenter with an anisotropic half-space:

(19) $$S=\frac{2}{\sqrt{\pi }}M\sqrt{A}$$

where

(20) $$M={\left[\frac{1}{2}\oint_{\xi_{1}^{2}+\xi_{2}^{2}=1}W\left(\xi_{1},\xi_{2}\right)\;ds\right]}^{-1}$$

The indentation modulus *M* depends on $W\left(\xi_{1},\xi_{2}\right)$, which is the Fourier transform of the normal displacement of the surface of the anisotropic half-space under the influence of a point load applied perpendicular to the surface.

It is worth noting that the contact stiffnesses (Equation (19)) take a form similar to that obtained for the spherical indenter and an isotropic medium (Equation (14)). The only difference is that the indentation modulus *M* depends, via the integral (Equation (20)), on both the indenter shape and the orientation of the tested material relative to the indentation axis. This means that the experimentally determined indentation modulus and hardness will vary depending on the crystallographic signature of the indented crystal surface.

Indeed, the results of indentation of the (100), (110), and (111) surfaces of Si single crystal with conospherical diamond indenter (tip radius of 5 μm, loading rate 5 mN/s) confirmed the above considereations (Gerbig et al. [23]). The determined values of the elastic modulus were 125 GPa for Si(001), 163 GPa for Si(110), and 180 GPa for Si(111). In another work by Tian et al. [24], the effect of strain rate on the elastic modulus of Si indentation was presented. The indentation modulus of single-crystal silicon exhibits small changes with respect to varying strain rates. The average *M* moduli obtained for the <100>, <110>, and <111> orientations were 163 GPa, 167 GPa, and 177 GPa, respectively. Indentations performed by Zhang et al. [25] on the (100), (110), and (111) surfaces of Si-doped GaAs crystals yielded qualitatively similar results. The determined values of the indentation modulus were $M_{\left(100\right)}=129$ GPa, $M_{\left(110\right)}=132$ GPa, and $M_{\left(111\right)}=139$ GPa. All presented data show a clear anisotropy of the mechanical properties of GaAs and Si crystals.

### 2.3. Singularities on the P(h) Curve

Due to the discrete nature of the plastic deformation, a smooth P(h) curve can exhibit discontinuities. When recorded during the loading stage–load-controlled regime: *P* applied, *h* measured—the discontinuity (pop-in) signals a sudden displacement of the indenter into the crystal at a very narrow interval of load. During operation in the displacement-controlled mode (*h* applied, *P* measured), the occurrence of pop-in is equivalent to reducing the force sensed by the indenter at a very narrow displacement range. Usually, the first pop-in event indicates the beginning of the nanoindentation-induced plastic deformation [26].

Association of the *pop-in* with dislocation nucleation is understandable as the nanoindentation allows for studying the crystal’s mechanical response in a subsurface nanovolume free of linear defects. Indeed, the dislocation density in annealed metal single crystals can reach values of the order of $10^{7}{\mathrm{cm}}^{-2}$, which gives an approximate interdislocation distance of ∼3 μm [27]. In GaAs single crystals obtained by the Czochralski method, the dislocation density is even lower, $10^{4}{\mathrm{cm}}^{-2}$ [28], resulting in an interdislocation distance of ∼100 μm. Since nanoindentation can be performed using indenters with a tip radius of several hundred or even several dozen nm, the nanoindentation in a dislocation-free region is quite likely.

According to the classical theory of dislocations [29], the shear stress required to form a dislocation loop of radius *r* can be determined from the equilibrium condition between the elastic energy of the dislocation loop and the work required to form it:

(21) $$\tau =\frac{2-\nu }{1-\nu }\frac{Gb}{4\pi r}\left[\ln\left(\frac{4r}{r_{0}}\right)-2\right]$$

where *G* and ν are the shear modulus and Poisson’s ratio, *b* is the length of the Burgers vector of dislocation, and $r_{0}$ is the radius of the dislocation core (it is assumed that $r_{0}=b/3$). A dislocation line segment’s character is determined by the orientation of its tangent vector relative to the Burgers vector. If the tangent vector is parallel to the Burgers vector, the segment is a screw dislocation. If the tangent vector is perpendicular to the Burgers vector, it is an edge dislocation. Dislocation loops often have segments with a mixed character, where the tangent vector is neither parallel nor perpendicular to the Burgers vector.

The maximum of the function τ=τ(r) gives the critical stress $\tau_{c}$ and the critical radius $r_{c}$ for the dislocation loop nucleation:

(22) $$\begin{array}{ccc} r_{c} & = & \frac{1}{4}r_{0}e^{3}\\ \tau_{c} & = & \frac{Gb}{4\pi r_{c}}\frac{2-\nu }{1-\nu }\approx \frac{3G}{\pi e^{3}}\frac{2-\nu }{1-\nu }. \end{array}$$

where *e* is Euler’s number. For example, for Al, the elastic constants G=71 GPa, ν=0.345 [30], which gives $\tau_{c}=8.5$ GPa. This stress is easily achieved during nanoindentation.

One of the first observations of singularities on the P(h) curve was published in the work by Corcoran et al. [31], who presented the results of studies on plastic deformation of Au single crystals with different crystallographic orientations. The recorded *pop-ins* were separated from each other by fragments of the P(h) curve, which reflected the elastic deformation of the crystal. Furthermore, an analysis of the results of experiments proved the hypothesis that discontinuities on the *P(h)* curve were caused by the generation of dislocations.

The *pop-in* does not have to be associated with forming linear defects. Indeed, sapphire is a strongly anisotropic crystal, and the mechanism of the onset of nanoindentation-induced plasticity depends on the orientation of the crystal surface relative to the indentation axis. Therefore, nanoindentation of the M-type surface caused the twinning in the base plane (C). In the case of nanoindentation of the A plane, a slip in the C plane [3,32] is responsible for incipient plasticity.

Another cause of discontinuities on the P(h) curve during loading is the structural phase transformation in the tested crystal [4]. The *P(h)* curve can also exhibit a singularity during unloading, as exemplified by the results of silicon nanoindentation. In this case, the so-called *pop-out* is caused by an increase in the volume of the unit cell, which occurs during the Si-II → Si-XII/Si-III [1] phase transition.

## 3. Silicon and Gallium Arsenide

About 50 years ago, stress-induced metallization of silicon and germanium, caused by a transformation from a semiconductor phase, was observed [33,34]. Since the first high-pressure semiconductor phases appear at pressures up to 20 GPa, both the generation of dislocations and structural phase transformations can influence nanoindentation-induced plastic deformation of Si and GaAs.

This review section will discuss the issues related to phase transformations in Si and GaAs. Essential information on dislocations in stressed semiconductor crystals and relevant references can be found in the review article by Yonenaga [35].

### 3.1. Structure and Phase Transformations In Silicon

**Figure 4:**
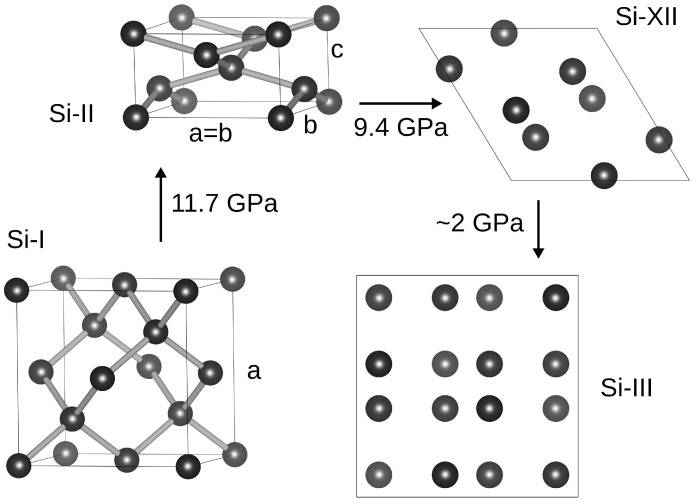
Unit cells of the phases: Si-I (A4), Si-II (β-Sn), Si-XII (R8), and Si-III (BC8). The latter two structures are shown in the projection onto the (001) plane.

Silicon, at ambient conditions, exhibit the A4 (cubic diamond, *cd*, Si-I, $Fd\bar{3}m$ space group) crystallographic structure with a lattice constant of a=5.431Å [36] (Figure 4). The Si-I phase is a semiconductor with an indirect band gap: $\Delta E_{g}=1.12\mathrm{eV}$ [37].

Under the influence of compressive stresses of ∼11 GPa (11.3 GPa [38], 11.7 GPa [39]), the Si-I phase transforms into the Si-II phase (Figure 4) with the structure of the β-Sn type and the $I4_{1}/amd$ space group symmetry. Silicon atoms occupy 4(a) Wyckoff positions with coordinates (0,0,0) and (0,0.5,0.25). The unit cell parameters a=b=4.665Å and c=2.565Å were measured at pressure 11.7 GPa [39]. The volume reduction of 21% accompanies this transformation [39]. The Si-II phase displays the metallic electrical conductivity [40].

In the pressure range from 13 to 16 GPa, the Si-II phase undergoes the transformation to the hexagonal Si-V phase (P6/mmm space group) with the silicon atom located at 1(a) Wyckoff position [36,41]. The unit cell parameters of the Si-V phase under pressure 16.3 GPa are: a=2.549Å and c=2.383Å [39].

New silicon crystal structures were discovered during the pressure relaxation from the Si-II phase. The complete removal of the applied compressive stress does not restore the initial Si-I phase. Instead, Si-III silicon is formed (Figure 4) with a BC8-type lattice ($Ia\bar{3}$ space group). Silicon atoms are located in the Wyckoff position 16(c) with coordinates (u≈0.15,u,u) [36]. The Si-II → Si-III transformation does not occur directly. After reducing the pressure to ∼9.4 GPa, a transformation to the Si-XII phase (R8 lattice type, $R\bar{3}$ space group). Silicon atoms occupy the Wyckoff positions (r-axes) 2(c), 6(f) with coordinates (w,w,w) and (x,y,z). The transformation is accompanied by an increase in volume by 10.7%. Lattice parameters of the Si-XII phase measured at the pressure of 6.3 GPa are: $a=5.63,\alpha =110^{\circ },w=0.2836,x=0.0.4620,y=-0.032,z=0.2667$ [36]. Further reduction in the compressive stress to ∼2 GPa causes the Si-XII → Si-III transformation with an increase in volume by 2.1% [36].

The Si-III phase was also synthesized under non-hydrostatic pressure. In work by Butler et al. [42], the results of the relevant experiments were demonstrated, and it was shown that the Si-III phase formed by nonhydrostatic compression to 20 GPa exhibits a strong preferred orientation, in which the crystals arrange themselves in the ⟨110⟩ directions perpendicular to the compression axis. The source of the preferred orientation was found to be the highly anisotropic Young’s modulus of the *sh* phase, which is transferred to subsequent (during stress relaxation) daughter phases via displacive phase transformations.

In the further part of the monograph, due to the stress level achieved during nanoindentation, the Si-I → Si-II and Si-II → Si-XII/III transformations will be of interest. Details about silicon phase transformations, which are not presented here, can be found in the works of [36,43,44]. A comprehensive and highly recommended study of silicon allotropes was published by Mujica et al. [41] and Fan et al. [45]

### 3.2. Structure and Phase Transformations in Gallium Arsenide

**Figure 5:**
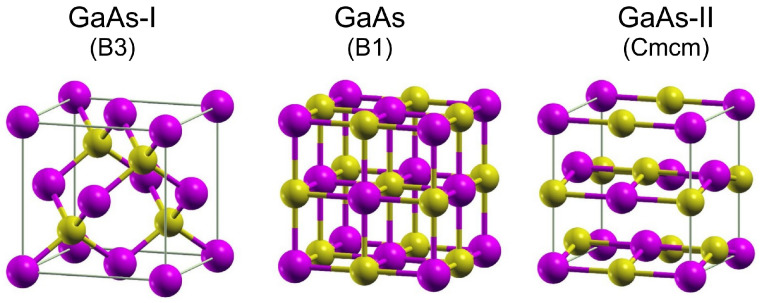
Unit cells of GaAs-I, B3 and high-pressure GaAs-II [46] phases. The unit cell of B1 GaAs was provided for the sake of comparison with the GaAs-II unit cell.

Under ambient conditions, gallium arsenide has a B3-type structure (GaAs-I, zinc blende). It is composed of two cubic diamond sublattices (a=5.6532Å) occupied by arsenic and gallium atoms, respectively, which are shifted relative to each other by the [0.25,0.25,0.25] vector [36]. The GaAs-I phase (Figure 5) belongs to the $F\bar{4}3m$ space group and is the semiconductor with the direct energy gap $\Delta E_{g}=1.42\mathrm{eV}$ [37].

The influence of compressive stresses causes a series of phase transformations to occur in the GaAs crystal. Investigations presented in the work of Weir et al. [47] showed that the GaAs-I single crystal at the pressure 16.6 GPa transforms into the GaAs-II phase with the symmetry defined by the space group Pmm2. The unit cell parameters of the GaAs-II phase were measured at the pressure 22.9 GPa: a=2.482Å, b=2.618Å, c=4.83Å. Ga and As atoms were located at the Wyckoff positions 1(a) and 1(c) with coordinates: (0,0,0) and (0,0.5,0.35), respectively.

However, Zhang et al. [46] performed DFT (Density Functional Theory) calculations, pointing to another possibility for GaAs. They found that the GaAs-II lattice exhibits Cmcm space group symmetry with atoms in the unit cell located at 4(c) Wyckoff sites: $\left(0,1/4,y_{Ga}=0.68\right)$, $\left(0,1/4,y_{As}=0.18\right)$. Thus, the defined GaAs-II phase lattice (Figure 5) has a symmetry described by the space group Cmcm. In the work of [36], it was shown that the unit cell of the structure with Cmcm symmetry, under the pressure ∼18GPa, is characterized by the lattice constants a=4.971Å,b=5.272Å,c=4.779Å and the location of Ga and As atoms in the Wyckoff positions 4(c) with coordinates (0,0.649,0.25) and (0,0.166,0.25), respectively. The GaAs-I → GaAs-II transformation is associated with a volume reduction by 14.3% [41,48]. The similarity of the crystal lattice of the GaAs-II phase to the NaCl-type structure justifies the term *rock-salt-like* often found in the literature. The GaAs-II phase is a metallic phase [49].

The next transformation, to the GaAs-III phase, occurs at a pressure of 24 GPa [47]. The symmetry of the GaAs-III phase is described by the space group Imm2. The crystal lattice of this phase, at pressure 28.1GPa, can be represented as orthorhombic (a=4.92Å, b=4.79Å, c=2.635Å) with Ga and As atoms in positions 2(a) with coordinates Ga(0,0,0) and As(0,0.25,0.425) [47].

Phase transformations of GaAs-I ↔ GaAs-II were the subject of detailed experimental studies conducted by Besson et al. [50]. The analysis of structural changes during the increase in compressive stresses to 22GPa confirmed the results of Weir’s work et al. [47]: at pressures of about 16GPa, the transformation into the GaAs-II phase begins. However, from about 12GPa, the instability of the GaAs-I phase was observed. The course of phase transformations during the relaxation of compressive stresses was also studied. After complete decompression, a mixture of the amorphous phase α-GaAs and GaAs-I was observed in the final state, but only in those single crystals that were not completely transformed to the GaAs-II phase. On the other hand, for samples whose structure was fully transformed to the GaAs-II phase, pressure relaxation caused the restoration of the GaAs-I phase without the participation of the amorphous phase. It should be added that the amorphization of polycrystalline GaAs has been observed earlier in samples subjected to rapid decompression from ∼115 GPa [51].

More recent publications do not fundamentally change the above picture of the GaAs-I → GaAs-II phase transition. For example, the paper by Zhang et al. [52] presents the results of studies of the GaAs structure performed up to 24.3 GPa under hydrostatic conditions and combined with in situ Raman spectroscopy and electrical conductivity measurements. During compression, a phase transition from the zinc blende (GaAs-I) to the orthorhombic GaAs-II (Cmcm) structure was observed at 12.2 GPa via discontinuous changes in the Raman shift and electrical conductivity. The results of electrical conductivity experiments at variable temperature confirmed that the high-pressure GaAs-II phase exhibited metallic behavior. After decompression, Raman scattering results of the recovered sample under ambient conditions indicated that the phase transition was reversible under hydrostatic conditions. The reversibility of the phase transition was further confirmed by HRTEM images of the recovered sample.

Additional information concerning structural phase transformations can be found in review articles [36,41]. In the further part of the work, special attention will be paid to the GaAs-I → GaAs-II transformation, because the level of nanoindentation stress is sufficient to initiate it and can be easily achieved during nanoindentation.

## 4. Nanoindentation of Silicon and Gallium Arsenide

With the development of the nanoindentation method and complementary methods of classical molecular dynamics, it has become possible to study the initial stage of plastic deformation in semiconductor crystals free of or with a low density of primary dislocations. Under the conditions defined by the stress field generated during nanoindentation, dislocation nucleation and structural phase transformations are the physical processes that affect the course of incipient plasticity.

### 4.1. The Lorentz–Leipner Criterion

The non-zero components of the stress tensor at the points located on the indentation axis *z* are given [11] by the following equations:

(23) $$\begin{array}{ccc} \frac{\sigma_{rr}}{p_{0}}=\frac{\sigma_{\theta \theta }}{p_{0}} & = & -\left(1+\nu \right)\left\{1-\left(z/a\right)\tan^{-1}\left(a/z\right)\right\}+\left(1/2\right){\left(1+z^{2}/a^{2}\right)}^{-1}\\ \frac{\sigma_{zz}}{p_{0}} & = & -{\left(1+z^{2}/a^{2}\right)}^{-1}. \end{array}$$

**Figure 6:**
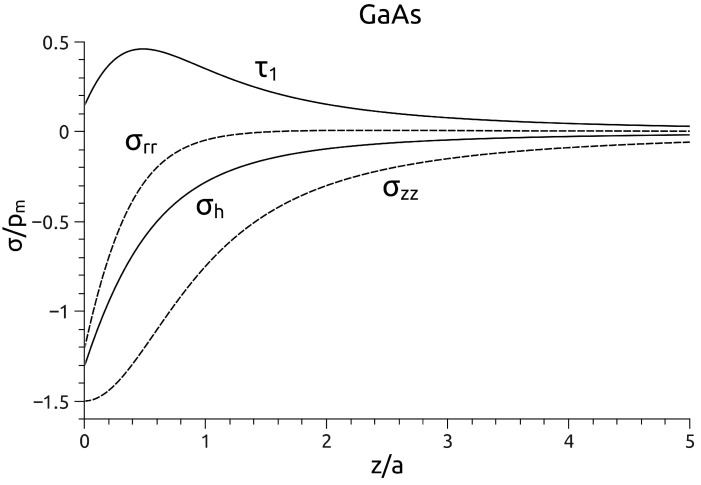
Stress distribution $\sigma_{zz}$ and $\sigma_{rr}$ along the indentation axis for GaAs. Maximum compressive stress $\sigma_{h}$ occurs at the point of contact between the surface and the indenter (z/a=0). The maximum shear (Tresca) stress $\tau_{1}$ reaches its extreme value at a certain distance from the indenter (z/a=0.49).

For GaAs, assuming ν=0.31, the maximum shear stress $\tau_{1}=\left(1/2\right)\left|\sigma_{z}-\sigma_{r}\right|$ reaches the value:

(24) $${\left(\tau_{1}\right)}_{max}=0.462\frac{P}{\pi a^{2}}=0.462p_{m}$$

at z=0.49a, whilst the hydrostatic pressure reaches the value:

(25) $${\left(\sigma_{h}\right)}_{max}=1.31p_{m}$$

at the contact surface (Figure 6).

It follows that the ratio of hydrostatic pressure to maximum shear stress assumes a constant value:

(26) $$\frac{{\left(\sigma_{h}\right)}_{max}}{{\left(\tau_{1}\right)}_{max}}=K$$

where K=2.84 for GaAs. This conclusion, derived from Hertzian theory of the elastic contact, allowed us to establish a criterion for approximately identifying which process: dislocation nucleation or structural phase transformation, will initiate plastic deformation during nanoindentation [53,54]. Suppose the ratio of hydrostatic pressure values at which a phase transformation occurs to the shear stress necessary for dislocation nucleation is less than *K*. In that case, plastic deformation can be expected to be initiated by a phase transformation. When this ratio is greater than *K*, dislocation nucleation is more likely.

### 4.2. Silicon

During nanoindentation of silicon, Si-I → Si-II phase transformation occurs. In the early work by Domnich et al. [55], the Raman spectroscopy method was used after nanoindentation of the Si(001) single-crystal surface (Berkovich indenter) to study the structure of the residual impression (refer to Figures 1 and 2 in [55]). The presence of Si-III, Si-XII, and amorphous (*a*-Si) phases in the plastically deformed volume was demonstrated. As proposed, it could only be caused by the Si-I → Si-II transformation during loading. Nanoindentations into the (001) surface of the Si crystal were performed with loading/unloading rate dP/dt from 1mN/s to 3mN/s. The obtained P(h) curves exhibited singularities only in the part corresponding to unloading. The initial stage of unloading is a partial relaxation of elastic deformation. For a smaller unloading rate, the *pop-out* event (discontinuity *P(h)*) was observed; however, for a larger unloading rate, the characteristic *elbow* (discontinuity dP/dh) perturbed the P(h) curve. In the first case, there is a sudden decrease in the crystal volume localized under the indenter, while in the second case, the volume change is continuous. Interestingly, when *pop-out* was observed, Raman studies showed the presence of dislocations and Si-III, Si-XII phases in the structure of the permanently deformed Si crystal. On the other hand, dislocations and the amorphous silicon were present when *elbow* was observed. *Pop-out* was recorded in the range of contact pressures from 5 to 8.5 GPa while *elbow* under pressures lower than 4.5GPa. For intermediate values of dP/dt, both singularities were observed (contact pressure of 4.5–5 GPa) accompanied by the presence of dislocations, Si-XII, Si-III, and *a*-Si phases in the plastic zone.

Structural changes occurring during silicon nanoindentation were also studied in the work by Juliano et al. [56]. They studied mechanical response of the (111) plane of Si crystal using a sphero-conical diamond indenter with a 90° included angle and effective tip radius of 13.5 μm. A nanoindentation test confirmed the course of the phase transformations described above. Specifically, the Si-I phase was found to be stable in the contact pressure range up to 10.5 GPa and over a range of applied maximum loads (25–700 mN), as well as loading/unloading rates (1–30 mN/s). The transformation to the high-pressure Si-II phase then started. It was observed that the Si-II phase was stable during unloading to approximately 3.5 GPa. For contact pressures less than 3.5 GPa, the experiments showed disturbances of the unloading P(h) curve, which resulted in the presence of crystalline Si-III/Si-XII phases as well as an amorphous one.

**Figure 7:**
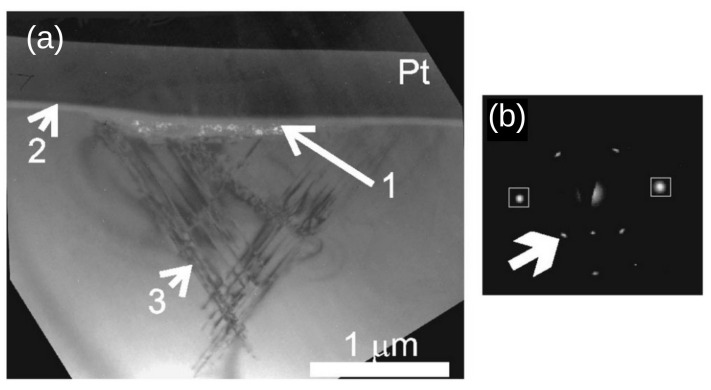
(**a**) Cross-sectional electron microscopy (XTEM) image of a spherical indentation in Si(001) crystal with a maximum load of 20 mN showing a polycrystalline layer of Si-XII phase (region 1). (**b**) Selected area diffraction pattern of polycrystalline region. Boxed diffraction spots are from (220) Si-I. All unboxed spots are from the polycrystalline Si-XII. The dark field image in (**a**) was taken using an arrow Si-XII spot in (**b**). Region 2 indicates amorphous Si while traces of slip planes are visible in region 3. Adapted with permission from Ref. [57].

**Figure 8:**
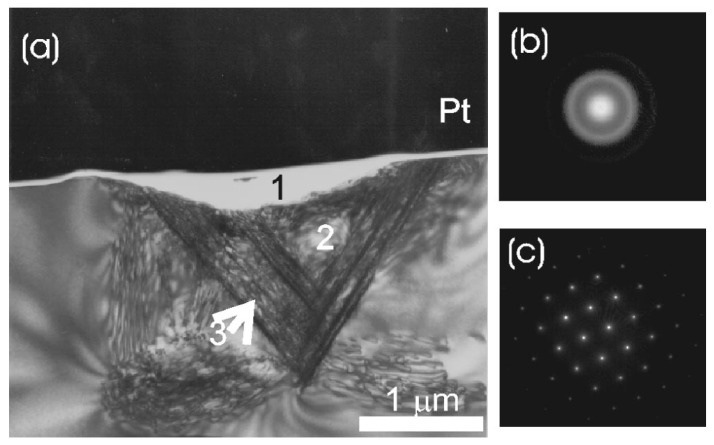
(**a**) Bright field XTEM image of spherical indentation in Si(001) crystal with a maximum load of 80 mN. (**b**) Diffraction pattern of amorphous Si taken from region 1 (directly under the residual indent impression). (**c**) Diffraction pattern of crystalline Si-I taken from region 2. Adapted with permission from Ref. [57].

Similar conclusions were posted in the works [57,58,59], where nanoindentation experiments were supplemented with structural investigations (the transmission electron microscopy (TEM), the Raman spectroscopy) of the silicon crystal after unloading. For example, the article by Bradby et al. [57] describes nanoindentation experiments performed on the (001) surface of a Si crystal with a spherical indenter (radius of 4.5 μm). Application of a maximum load of 20 mN produced a complex microstructure in the vicinity of the residual impression (Figure 7). The following were observed: (1) a thin amorphous Si layer directly under the indenter with small particles of the Si-XII crystal phase embedded in the amorphous one, and (2) slip bands indicating dislocation activity during plastic deformation. Application of a larger maximum load of 80 mN resulted (Figure 8) in the presence of an amorphous Si layer and slip bands located beneath it (region 3).

The data discussed above indicate that plastic deformation of silicon crystal during nanoindentation is a complex process governed by dislocation activity and structural phase transformations. The effect of the Si-I → Si-II transformation on the plastic deformation during loading is confirmed (indirectly) by the occurrence of some singularities on the *P(h)* unloading curve: the *pop-out* event indicating the beginning of the Si-II → Si-XII/III phase transformations and the *elbow* event which signals the beginning of the Si-II → *a*-Si transition.

In light of the presented results, the following question arises—what process initiates the plastic deformation of free dislocations in Si crystals during nanoindentation: a structural phase transformation or the nucleation of the first dislocation? As is known, the formation of the first linear defects is accompanied by the *pop-in* discontinuity on the P(h) curve. However, it happens not only for metals (e.g., [31]), but also in crystals with covalent bonds such as GaN [60], for which the first phase transformation occurs at the hydrostatic pressure of approximately 45 GPa [61].

Assuming that the absence of *pop-in* on the loading curve of Si crystal suggests that a dislocation nucleation process does not initiate the plastic deformation, the occurrence of the Si-I → Si-II phase transformation was considered. But the mentioned phase transition is burdened with a significant reduction in the volume of the unit cell, and therefore, pop-in was expected as a sign of this transition. The absence of *pop-in*, and thus the gradual course of the loading part of the *P(h)* curve, can be explained by the “geometric similarity” of the Si-I and Si-II unit cells. As shown in the work of Kim et al. [62], parameters (a=b,c) of the Si-I unit cell in tetragonal representation changed gradually during loading (refer to Figure 2 in [62]), ultimately taking the values characteristic for the Si-II phase.

Further arguments in favor of the assumption that nanoindentation-induced plastic deformation in silicon starts with a phase transformation were provided by Gerbig et al. [23]. To determine the beginning of plastic deformation, the authors of this work used the multiple partial unloading technique: the maximum load is not reached directly, but through a sequence of “loading–partial unloading” cycles. Consequently, the P(h) curve is contained between two envelopes. The point at which the envelopes begin to diverge will indicate the onset of plasticity. It was found that plastic deformation occurs at contact pressures of 12GPa, 10.5GPa, and 8.8GPa for nanoindentation of the (001), (110), and (111) Si surfaces, respectively. The measured contact pressures at the onset of plastic deformation were consistent with the hydrostatic pressure at which the Si-I → Si-II transformation occurs (∼11 GPa).

Incidentally, *pop-in* is observed on the P(h) curve. However, the results of works, for example, Bradby et al. [63] and Gerbig et al. [23], revealed that this effect is not related to the onset of plastic deformation, but to a slightly later extrusion of the Si-II phase from under the indenter onto the crystal surface.

Results of Gerbig et al. [4,64,65,66] provided new knowledge about the course of phase transformations in silicon during nanoindentation. They utilized a unique measurement system consisting of an indentation head and the Raman spectrometer. A thin Si layer deposited on a sapphire wafer was subjected to spherical indentation. The optical system of the Raman spectrometer was placed along the indentation axis and on the opposite side of the wafer. Such construction enabled the authors to record Raman spectra with the load-displacement curves. During loading, the transformation from Si-I to Si-II phase was observed; however, the phenomenon was accompanied by the formation of another phase of silicon, recognized as BCT-5. The bct5 phase was earlier predicted by DFT calculations by Boyer et al. [67]. The Raman peak corresponding to the BCT-5 phase was recorded at the contact pressure of 6.2 GPa, while the transformation to the Si-II phase was ended at 11.2 GPa. Further, the Si-II phase was transformed into a mixture of Si-XII/Si-III phase during unloading. The measurements showed that the Si-XII phase can form simultaneously with the Si-III structure in contrast to the previous assumption of a two-step process: Si-II → Si-XII → Si-III.

Another system for synchronous measurements of indentation (Vickers indenter) and Raman spectroscopy was proposed by Wu et al. [68]. During indentation loading, a successive decrease in the Raman peak corresponding to the Si-I phase was observed, followed by the presence of the Si-II phase. During the unloading process, and in correlation with the occurrence of pop-out, the presence of a mixture of Si-III/XII phases was recorded on the p(h) curve, which is synchronized with the occurrence of the pop-out phenomenon. The initial fraction of Si-III/XII phases detected in the contact area was 12.5% and then it increased steadily until the end of indentation.

The existence of metastable phases of silicon is not only related to the unloading state of the silicon. Indeed, another experiment by Gerbig et al. [66] showed that the Raman spectrum of the indented Si crystal with a 50 mN load (Berkovitch indenter) and held for an hour-long period contains peaks identified as belonging to Si-II and other high-pressure phases. Detailed analysis confirmed that the transformation to high-pressure metastable phases of silicon (Si-XII, Si-III) started already during loading.

### 4.3. Gallium Arsenide

The P(h) curves recorded during nanoindentation of gallium arsenide and silicon differ. The elastic deformation of GaAs ends with *pop-in* [53,69,70,71,72], and further penetration of the indenter into the material causes its permanent deformation. TEM examinations of the cross-section of the residual cavity showed traces of slip bands {111}, highly developed dislocation networks, and a crack lying on the indentation axis [69,70,73]. The dislocation structure in the vicinity of the residual impression was of the form of a three-arm rosette (surface (111)) and a four-arm rosette (surface (001)) [71,74,75]. The shape of the P(h) curve during unloading was free of singularities.

In contrast to silicon, high-pressure phases of gallium arsenide were not observed beneath the residual cavity [69,76]. It was concluded that during nanoindentation, no phase transformations occur, or they do occur, but during unloading, a reverse transformation completely restores the parent phase. There are two publications [77,78] in which an amorphous GaAs phase was found to have formed in the vicinity of the residual impression as a consequence of the application of a very high unloading rate. This result is consistent with the previously presented results of phase transformation studies, when the increase in the hydrostatic pressure caused the GaAs-I → GaAs-II transformation, but rapid stress relaxation caused the amorphization of gallium arsenide [50,51]. Despite this, a literature review on GaAs crystal nanoindentation experiments indicates the dominant dislocation picture of the onset of plastic deformation. Therefore, it was assumed that the cause of the *pop-in* phenomena is the nucleation of dislocations.

The problem of the onset of nanoindentation-induced plasticity in semiconductor crystals was also discussed in the articles by Lorenz et al. [54], Leipner et al. [53], as well as Bradby et al. [70]. The authors of the first two papers, based on the classical theory of dislocations in a homogeneous and elastic medium, proposed a simple criterion for assessing whether a structural phase transformation can initiate plastic deformation in the crystal (refer to Section 4.1). For GaAs, the ratio of the maximum hydrostatic pressure to the maximum shear stress (or Tresca stress) satisfies the relation: ${\left(\sigma_{h}\right)}_{max}/{\left(\tau_{1}\right)}_{max}=2.84$. If the ratio of the hydrostatic pressure $\sigma_{PT}$ of the GaAs-I → GaAs-II phase transformation to the critical shear stress $\tau_{c}$ necessary for the formation of an equilibrium dislocation loop is greater than 2.84, then it is probable that plastic deformation will initiate the dislocation process, not the phase transformation.

The shear stress required to form a dislocation loop of radius *r* is described by Equations (18) and (19). The elastic constants of GaAs are: $c_{11}=119\mathrm{GPa}$, $c_{12}=53.4\mathrm{GPa}$ and $c_{44}=59.6\mathrm{GPa}$ [37]. Gallium arsenide is an anisotropic material, therefore the shear modulus *G* will be calculated with of the Voight method: $G=c_{44}-H/5=48.9\mathrm{GPa}$ where $H=2c_{44}+c_{12}-c_{11}=53.3\mathrm{GPa}$ [79]. In this approximation, Poisson’s ratio ν=0.31 [37] and, consequently, the critical stress $\tau_{c}=5.7\mathrm{GPa}$. Dividing, the pressure $\sigma_{PT}=16.6GPa$, under which the GaAs-I → GaAs-II transformation occurs [47,50] by $\tau_{c}$ gives: $\sigma_{PT}/\tau_{c}=2.9>2.84$. This result indicates that the origin of the elastic–plastic transition in GaAs crystal can be governed by the dislocation nucleation. However, considering the approximate nature of the Lorentz–Leipner criterion and the small difference 2.9−2.84, one cannot definitely reject the structural phase transformation as the mechanism initiating nanoindentation-induced plasticity in GaAs crystal.

In a similar analysis performed for Si, $c_{11}$ = 166 GPa, $c_{12}=64$ GPa, $c_{44}=79.6$ GPa G=68.1, ν=0.27 was assumed and $\tau_{c}=7.7\mathrm{GPa}$ was obtained. Furthermore, ${\left(\tau_{1}\right)}_{max}=0.476p_{m}$, ${\left(\sigma_{h}\right)}_{max}=1.27p_{m}$ and ${\left(\sigma_{h}\right)}_{max}/{\left(\tau_{1}\right)}_{max}=2.67$. Taking into account that the hydrostatic pressure under which the Si-I → Si-II transformation occurs is ∼11 GPa, the ratio $\sigma_{PT}/\tau_{c}=1.42<2.67$. Therefore, the expectation of a phase transformation scenario for the initial plasticity of the silicon crystal induced by nanoindentation is not without foundation.

The problem of the onset of plasticity in GaAs was the subject of computer simulations using the classical molecular dynamics method. Nanoindentations were performed with a cube of edge L=28Å [80]. The crystal was modeled using the Tersoff-type potential in the Albe parameterization [81], which allows for the correct description of both the elastic properties and structural parameters of several GaAs phases, including GaAs-I and GaAs-II. During nanoindentation, the volume located right beneath the cube was transformed into the GaAs-II phase, so it was possible to recognize the planes with an atomic arrangement similar to the B1 (rock salt) type structure. The simulated transformation was accompanied by a characteristic *pop-in* on the indentation curve.

Another example of molecular dynamics simulation of the GaAs-I → GaAs-II transformation can be found in the work by Rino et al. [82], where a single crystal of gallium arsenide was subjected to hydrostatic pressure. At the pressure of ∼18 GPa, the onset of the GaAs-I → GaAs-II transformation was noted, accompanied by a step change in the Ga-As bond length (see Figure 7a in [82]). This result, consistent with the experimental observations [50] (refer to Figure 7b [82]), shows an entirely different (step) nature of the GaAs-I → GaAs-II transformation compared to the Si-I → Si-II one, during which the change in lattice parameters is gradual (Figure 2 in [62]). These results indicate that pop-in during nanoindentation of a GaAs crystal can be caused not only by dislocation nucleation but also by a phase transformation.

From the data presented above, the mechanism of the onset of plasticity in nanoindented non-crystalline GaAs is unclear. Indeed, investigations by TEM and Raman spectroscopy indicated dislocation’s origin of the onset of plastic deformation. On the other hand, the results obtained by Li et al. [77,78] showing amorphization of gallium arsenide during fast unloading, the results of MD simulations, as well as the approximate outcome of the analysis based on the Lorentz–Leipner criterion suggest that the structural phase transformation can initiate the plastic deformation in nanoindented GaAs crystal.

**Figure 9:**
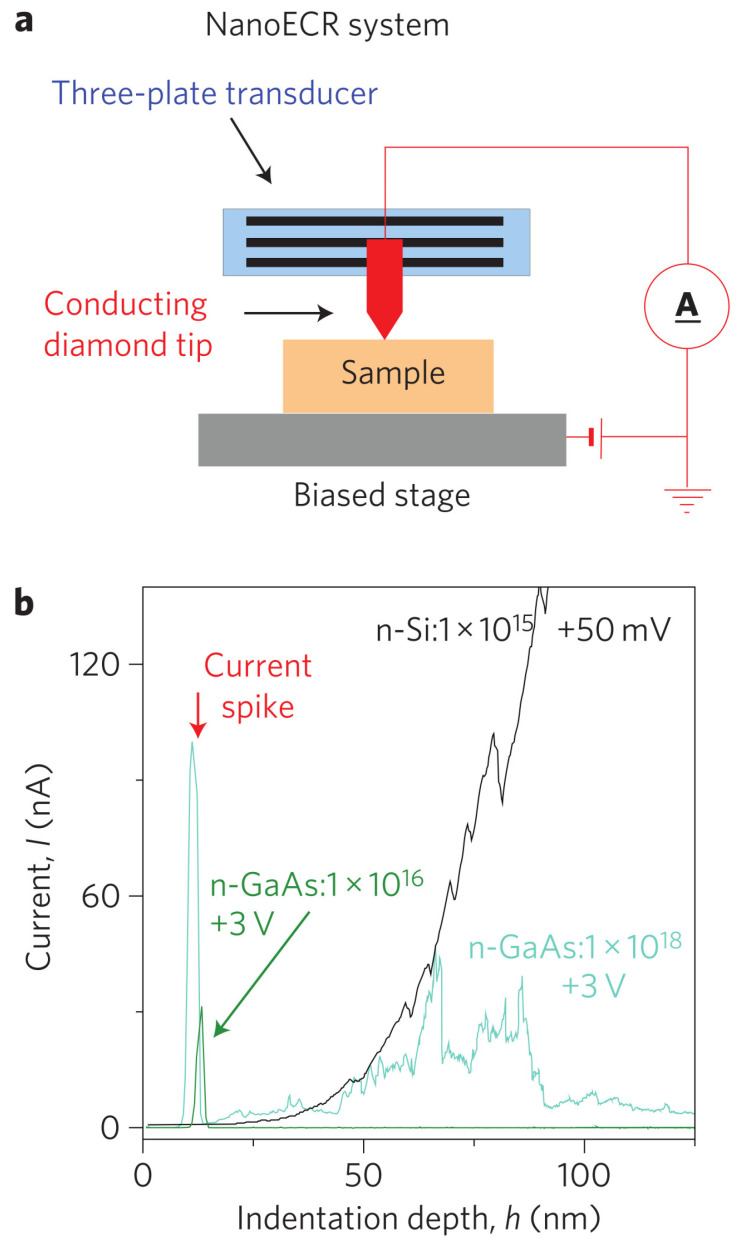
NanoCER measurements during nanoindentation. (**a**) Schematic of the measurement setup. (**b**) I(h) curves recorded for GaAs doped ($1\times 10^{16}$ and $1\times 10^{18}$${\mathrm{cm}}^{-3}$) and Si. Adapted with permission from Ref. [83].

**Figure 10:**
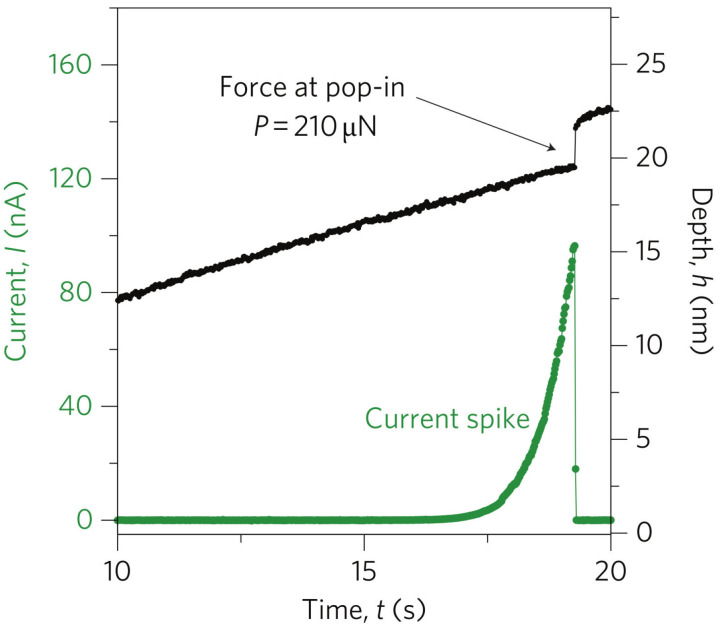
Typical result of the in situ electrical conductivity measurement for the nanoscale deformed GaAs crystal, which demonstrates the simultaneous occurrence of the current spike and the pop-in event during nanoindentation with a conductive tip. The measurements were carried out on a low-doped ($1\times 10^{16}$${\mathrm{cm}}^{-3}$) sample with a reverse bias of 5 V. Adapted with permission from Ref. [83].

Microscopic and mechanical studies did not indicate a specific mechanism of the onset of nanoindentation-induced plasticity in GaAs crystal. Since the electrical conductivity of the GaAs-II phase is much higher than that of the GaAs-I phase, the system measuring the electric current flowing in a system consisting of a conducting indenter and GaAs crystal surface was considered promising. The nanoECR system (Bruker/Hysitron) allowed measurements of the electrical conductivity of the contact between the conducting indenter (diamond highly doped by Boron) and the semiconductor’s surface. Experimental data presented in work by Nowak et al. [83] revealed a previously unknown electrical effect in Si-doped GaAs crystals, characterized by a pronounced current peak at the early deformation stage (Figure 9). The junction between the conducting indenter and the GaAs-I crystal surface, which can be classified as a Schottky contact, prevented (under the reverse bias) electrical current flow at the very initial stage of indentation. The observed leakage current through the junction started during elastic deformation. Furthermore, the electrical current increased rapidly and then a sudden electrical current drop to zero at the contact pressure of the mechanical pop-in event occurred (Figure 10). The phenomenon responsible for the pop-in event restores a rectifying nature of the contact between the conducting diamond indenter and the GaAs crystal.

This, so called, current-spike phenomenon contrasts with the expanded electrical response of Si crystal revealed by Nowak et al. [83] (Figure 9) and earlier by Ruffell et al. [2] who wrote that initial “rectifying characteristic originates from the contact between a highly doped indenter tip having almost metallic properties and the p-type silicon, thus creating a Schottky contact. At maximum load, a relatively good Ohmic characteristic is measured, indicating that potential barriers between the tip and metallic Si-II, as well as Si-II and the surrounding Si-I matrix, are not significant.”.

The observed combined pop-in and current-spike phenomena in GaAs cannot be attributed to dislocation activity but rather to GaAs-I → GaAs-II phase transformation. Indeed, the effect of GaAs crystal doping on dislocation formation during crystal growth, in detail investigated by Bourret et al. [28], shows that increasing the dopant concentration (silicon) reduces the possibility of dislocation nucleation. This effect is in contrast to the results of nanoECR experiments, which indicate an earlier (in a meaning of contact pressure) occurrence of the current spike for heavily doped ($1\times 10^{18}$${\mathrm{cm}}^{-3}$) GaAs at an average contact pressure of 16.8±0.1 GPa at the pop-in event, while lower doped samples ($1\times 10^{16}$${\mathrm{cm}}^{-3}$) require the pop-in contact pressure 18.4±0.4 GPa. If the pop-in were caused by dislocation activity in GaAs, the relationship between the pop-in contact pressure and the Si-doping level should be opposite. Therefore, the “downward” direction of the pop-in shift with Si-dopant concentration suggests that the current spike does not originate from dislocation nucleation.

**Figure 11:**
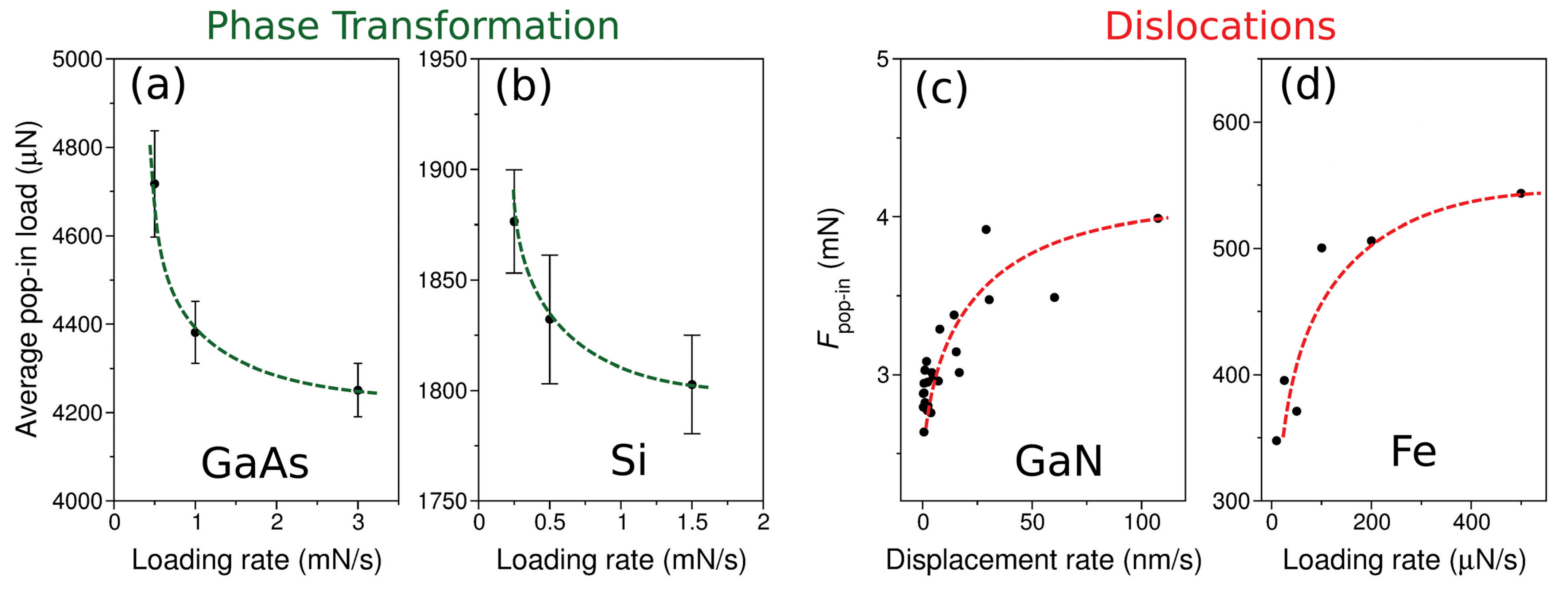
The results of nanoindentation experiments on GaAs (**a**) and silicon (**b**) represented by loading-rate dependence of the pop-in load. It contrasts the relationships recorded for (**c**) GaN [85] and (**d**) Fe [86]. Adapted with permission from Ref. [84].

This result was confirmed by a study of the effect of loading rate on the load at the pop-in event of GaAs and Si crystal [84]. The average pop-in load decreased with increased loading rate for both Si and GaAs crystals, contrasting the results of work by Fujikane et al. [85] and Gao [86] who studied incipient plasticity of GaN and Fe crystals, respectively. These authors observed that an increase in the loading rate increased the load at the pop-in event. Knowing that a mechanism of the incipient plasticity in GaN and Fe is related to the dislocation activity, one can conclude that incipient plasticity in GaAs is based on the phase-change scenario (Figure 11).

**Figure 12:**
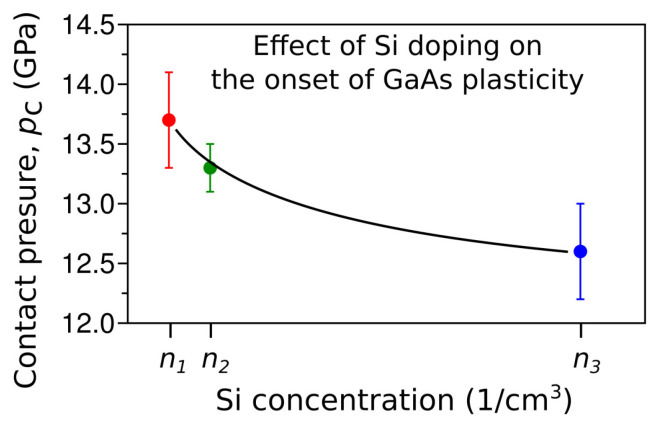
Results of the nanoindentation experiments performed for silicon Si-doped GaAs crystals. The relationship between the mean value of the pop-in contact pressure, pc, and Si concentration proves a slight decrease in the contact pressure at the pop-in. Adapted with permission from Ref. [87].

Further investigation of nanoindentation incipient plasticity of GaAs focused on the effect of doping on the contact pressure at the pop-in event. Particularly, the effect of Si doping was investigated by Chrobak et al. [87]. Nanoindentation experiments performed for Si-doped GaAs crystals ($n_{1}=5.5\times 10^{16}$, $n_{2}=3.1\times 10^{17}$, and $n_{3}=2.6\times 10^{18}$ cm^−3^) showed a decrease in the contact pressure at the pop-in with increasing dose of dopant atoms (Figure 12). In order to shed light on that result, first-principle DFT calculations were performed. The main goal of these simulations was to study the influence of silicon atoms on the equilibrium pressure between GaAs-I and GaAs-II phases. Obtained results showed a decrease in the equilibrium pressure due to doping. Consequently, this outcome suggested a phase transformation origin of the nanoindentation-induced incipient plasticity in GaAs crystal.

**Figure 13:**
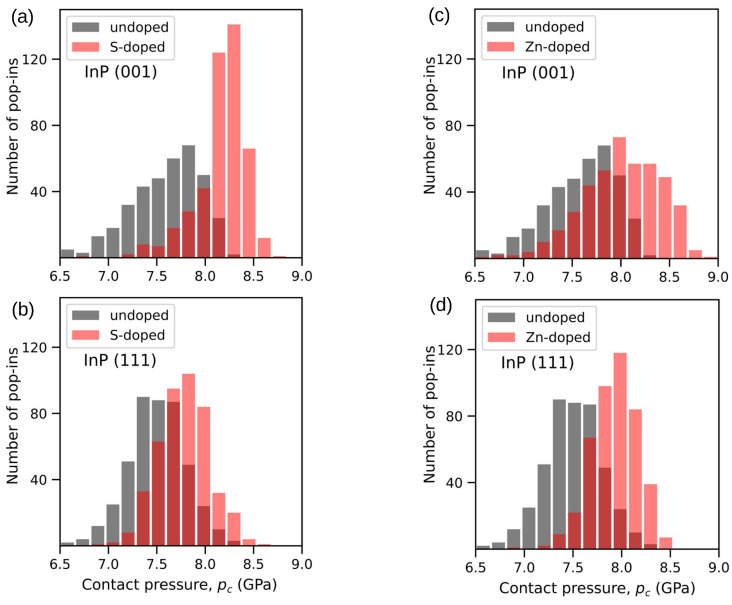
The histogram analysis showing the relationship between the number of pop-in events and their contact pressure $p_{c}$. (left column) Indentations were performed along the (**a**) [001] and (**b**) [111] crystallographic axes of the undoped as well as S-doped InP crystal. (right column) For Zn-doped InP crystal, indentations were also performed along the (**c**) [001] and (**d**) [111] crystallographic axes. Adapted with permission from Ref. [87].

A comparative study of nanoindentation-induced elastic–plastic transition was presented in work by Chrobak et al. [87], where nanoindentation experiments and DFT calculations were performed for GaAs and InP crystal. In contrast to the case of GaAs crystal, doping by Zn and S atoms caused an increase in the pop-in contact pressure distributions (Figure 13), which indicated the mechanism of nanoindentation-induced incipient plasticity governed by dislocation nucleation.

**Figure 14:**
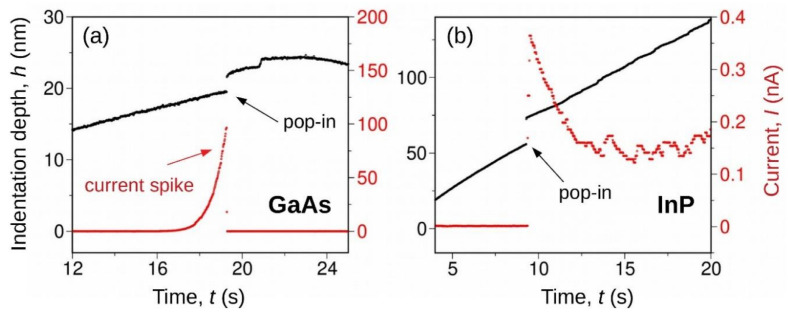
nanoECR experiments on GaAs and InP crystals. (**a**) The pop-in event stops the electrical current in GaAs. (**b**) The pop-in event initiates the electrical current for InP. Adapted with permission from Ref. [88].

The shift in the pop-in contact pressure distributions observed for GaAs and InP crystals is not the only indication of the different nature (mechanism) of the elastoplastic transition during nanoindentation. The nanoECR measurement results presented in Chrobak et al. [88] demonstrate fundamental differences in the dependence of the electric current I(t) flowing through the reverse-biased indenter-semiconductor surface junction (Figure 14). In the case of the GaAs crystal, we are dealing with the well-known current spike effect [83]. In contrast, the current response of the indented InP crystal is characterized by a zero value until pop-in occurs. Then, a sudden increase in electric current intensity is observed. This “unblocking” of the metal-semiconductor junction has been linked to dislocation nucleation at the moment of pop-in.

### 4.4. Molecular Dynamics Simulations

Classical molecular dynamics (MD) allows modeling physical phenomena at the atomic level. The volume affected by the nanoindentation stress is small and difficult to access by experimental techniques developed for structural investigations; therefore, MD simulations often assist the analysis of nanoindentation results. The MD method is based on numerical solution of the classical atomic equations of motion, also providing sophisticated procedures for controlling the number of particles, the system’s temperature, the total energy, as well as the pressure applied to the simulation box [89,90]. A key element of the MD method is calculating the force of interatomic interactions. Significant effort has been devoted to ensuring the physical meaning and developing parameterized mathematical models of the interaction potential energy in covalent crystals. A potential energy of multi-particle systems such as the Stillinger–Weber (SW) [91] contains, in addition to the two-body potential term, a three-body term stabilizing the tetragonal bond angle of $109.5^{\circ }$. Another method is based on modifying the attractive two-body part of the interaction potential energy by a so-called bond order function, which considers the effect of the environment on the strength of the two-atom interaction. Such potentials were proposed in the works of Tersoff et al. [92,93,94,95], Kumagai et al. [96], Albe et al. [81], Erhart et al. [97], Bazant et al. [98]. Furthermore, the potential developed by Pastewka et al. [99] correctly describes the brittle behavior of the Si-C system. Although most potentials accurately model the fundamental physical properties of semiconductor crystals—lattice parameters, the cohesion energy, elastic constants, and point defect formation energies—simulations of structural phase transitions still pose a significant challenge [100]. Currently, great promise is placed in force fields generated using machine learning methods [101,102].

In the work of Kim et al. [62], the contact of the (001) silicon crystal surface with a spherical indenter was studied. Simulations were performed at 300 K using the Tersoff potential [95]. The presence of atoms constituting BCT-5 and Si-II phases of silicon was observed during loading. The formation of the Si-II phase preceded the appearance of the BCT-5 one, which is interesting, however, inconsistent with the results of Gerbig et al. [4], who observed (using the Raman spectroscopy) the reverse sequence of transformations: BCT-5 → Si-II. Additionally, no dislocations were observed in the deformed silicon crystal. The lack of dislocations is, unfortunately, a trademark of Tersoff-type potentials. Conversely, the SW potential models properties of dislocations in Si quite well [103,104,105] as well as amorphous silicon. However, due to its design, it cannot describe transformations among high-pressure phases. These differences are well illustrated by examples of modeling the plasticity in compressed Si nanospheres. In the study by Valentini et al. [106], which used the Tersoff potential [93], the Si-I → Si-II phase transition was observed around the center of the nanosphere. In contrast, the work by Chrobak et al. [107] demonstrated that the SW potential enabled modeling of dislocation nucleation, their expansion, and ultimately the formation of steps on the nanosphere surface.

Although the course of computer simulations fundamentally depends on the choice of the interaction potential, the results presented in many other works give a picture of the sequence of phase transformations induced by nanoindentation, similar to the one presented in the previous paragraphs. However, it is known from TEM investigations (e.g., [57]) of the residual impression structure that phase changes induced by nanoindentation are accompanied by the generation of linear defects. Selected works on modeling silicon nanoindentation involving dislocations will be briefly presented in the following paragraphs.

The results of nanoindentation modeling of single-crystal Si using the Tersoff-type potential modified by Kumagai et al. [96] were presented by Zhang et al. [108]. The nanoindentation-induced Si-I → BCT-5 was initiated in the {111} planes of the Si-I phase, followed by amorphization of Si in the transformed volume. Moreover, the formation of perfect 1/2<110> dislocations and partial 1/6<112> dislocations from the boundary of the high-pressure phases was recorded. The dislocation line length recorded for the unloaded state (refer to Figure 8 in [108]) was generally smaller than one could expect from experiments (e.g., [57]).

Another paper worth citing in this review is the publication by Sun et al. [109]. To model changes in the structure of the indented silicon crystal, a modified bond-order potential proposed by Pastewka et al. [99] was used. Similar to the Raman spectroscopy measurements [4], a transformation from the Si-I phase to the Si-II phase followed by a transformation to the BCT-5 phase was observed. The BCT-5 structure surrounded the volume occupied by the Si-II phase. Continuation of the nanoindentation led to a gradual transformation of the high-pressure phases (Si-II, BCT-5) into amorphous silicon. Then, the amorphous phase extruded (continuously) outside the indentation cavity. During this process, but after the formation of the high-pressure phases, nucleation of the 1/2<110> dislocation was observed. Unfortunately, increased indentation stress did not significantly develop the dislocation line length (see Figure 7 in [109]).

Interesting computer simulation results were presented in the work of Sun et al. [110], in which the Pastewka potential [99] was used for interatomic interactions. The Si(001) surface was indented with spherical indenters with diameters ranging from 20 to 100 nm. It turned out that the Si-I → BCT-5/Si-II phase transition initiates plastic deformation of silicon, followed by the development of a dislocation network. The authors of the presented publication observed shifted dislocation loops in the 〈110〉{111} slip system emitted from the high-pressure phase/Si-I interface. The influence of the indenter diameter on the course of this complex plastic deformation process is significant. Namely, a large-diameter indenter promotes dislocation activity, while a small-diameter indenter is favorable for phase transitions.

**Figure 15:**
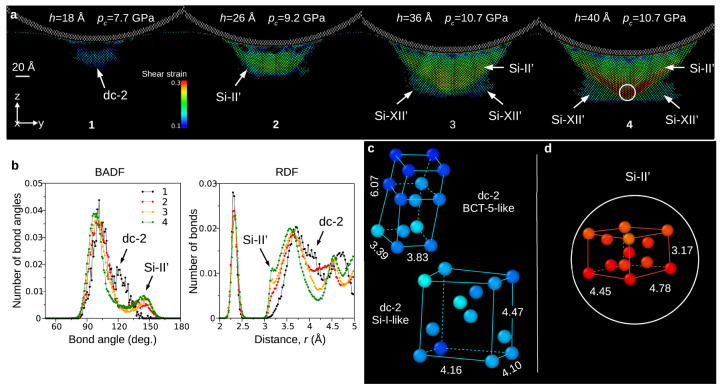
Indentation-induced phase transformations of the Si crystal modelled with the IP potential. (**a**) Structure evolution registered for the following indentation depths: h=18,26,36,40 Å. The atoms of the original Si-I phase are not displayed. The 20 Å thin sections of the transformed zone show the presence of dc-2/BCT-5 phase (snapshot 1), the Si-II phase (snapshots 2 and 3), and illustrate the formation of the Si-XII phase (snapshots 3 and 4), either directly under or in the vicinity of the Si-II volume. (**b**) BADF and RDF functions for snapshots 1-4, excluding the Si-XII structure. (**c**) The atomic arrangement in the detected dc-2/BCT-5 phase. The BCT-5 and dc-2 unit cells demonstrate alternative views of the same structure. (**d**) The Si-II unit cell. Adapted with permission from Ref. [111].

**Figure 16:**
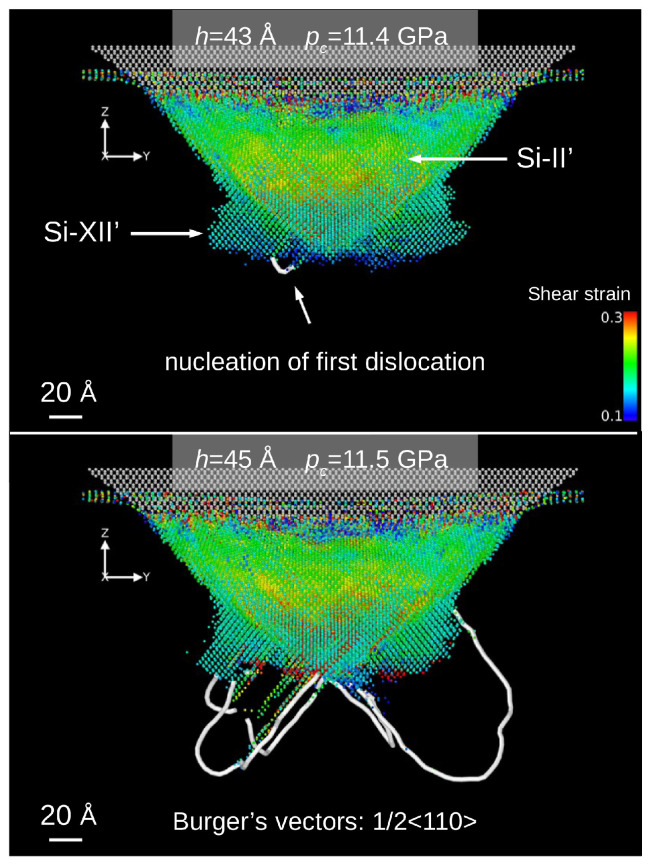
MD simulations with the IP potential revealed nucleation and the development of dislocations. The dislocation lines are situated in the 111 planes with the Burger’s vectors 1/2 <110>. Adapted with permission from Ref. [111].

**Figure 17:**
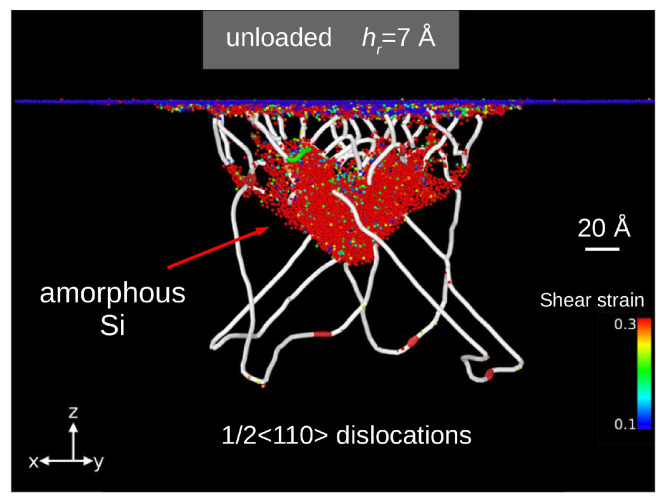
MD simulations with IP potential. The structure of the permanently deformed Si crystal obtained after complete unloading. Dislocations with Burger’s vector mainly 1/2 <110> (white curves) begin and terminate either in an amorphous phase (red atoms) or crystal surface. Adapted with permission from Ref. [111].

In the work by Abram et al. [111], a new integrated potential (IP) for silicon based on a hybridization of the SW [91] and Tersoff [93] potentials was proposed. A small addition of SW potential was intended to introduce an “element of stiffness” to the Tersoff model, thus enabling simulations of both phase transitions and dislocation nucleation. The undertaken computer simulations demonstrated a course of phase transitions consistent with the results of Raman studies [4]. Contact of a rigid spherical indenter with the (001) silicon crystal surface causes, in agreement with the Hertz theory of elastic contact, shear stress concentrations close to the crystal surface. Subsequently, the first Si-I → BCT-5 phase transition occurred. The designation of the first high-pressure structure appearing during nanoindentation as BCT-5 is ambiguous. As shown in Figure 15c, this non-diamond silicon phase can also be described as a highly deformed Si-I structure (dc-2). An increased applied stress caused the BCT-5 phase to transform into the Si-II phase, and a high-pressure silicon phase with a structure similar to Si-XII appeared beneath it (Figure 15). Finally, after the sequence of phase transitions is exhausted, dislocation nucleation starts. Further nanoindentation does not change the phase composition surrounding the indenter, but the dislocation expansion in the {111} planes was registered (Figure 16). Interestingly, during indenter unloading and due to the short duration of this process, the high-pressure phases transformed into amorphous Si, leaving a well-developed dislocation network (Figure 17). In particular, the final state of the Si crystal corresponds to many TEM studies revealing an amorphous phase and a dislocation network in the vicinity of the residual impression.

## 5. Conclusions and Perspectives

The problem of nanoindentation-induced incipient plasticity in GaAs has not received as much attention as it has for Si, where a phase transformation scenario was proved by in situ Raman spectroscopy measurements [4,20,65,66] and electrical conductivity measurements [63]. However, available data suggest that a structural phase transformation initiates nanoindentation-induced plastic deformation also in GaAs crystal. This hypothesis is confirmed by the results of early computer simulations, and above all by the results of nanoindentation experiments on doped GaAs, including those supplemented by nanoECR measurements (see Section 4.3). In this context, it’s worth noting the relationship between the contact pressure at the moment of pop-in and the doping level of GaAs and InP. Increasing the dopant concentration leads to a decrease in contact pressure for GaAs, while, conversely, it causes an increase for InP (Figure 12 and Figure 13). The presence of point defects in the crystal lattice inhibits the generation and development of dislocation networks and, by compromising the structural stability of the crystal, promotes the initiation of phase transition. This observation is consistent with the nanoECR results mentioned earlier. For GaAs, the pop-in, which involves a transition from semiconducting GaAs-I to a metallic GaAs-II phase, restores the metal-semiconductor contact in a metallic indenter/GaAs-II/GaAs-I system and, thereby, stops the flow of electric current in a reverse-biased system (Figure 10 and Figure 14a). In the case of InP, the occurrence of pop-in initiates the flow of electric current (Figure 14b), likely due to the generation of dislocations that provide a convenient path for the movement of electric charges. Furthermore, theoretical analysis based on the Lorentz–Leipner criterion does not contradict that GaAs-I → GaAs-II phase transformation initiates the elasto-plastic transition in GaAs crystal. To come closer to a complete understanding of the course of nanoindentation-induced plasticity of the GaAs, the current state of knowledge should be supplemented with Raman studies similar to those performed for Si [4,20,65,66]. MD computer simulations based on new interaction potentials with greater transferability than those currently known would help interpret new experimental results.

In the case of silicon, incipient plasticity is moderated by transformations from the Si-I to BCT-5 phase, and then to Si-II one. Other high-pressure phases (Si-II, Si-XII) and the dislocation nucleation are observed at a later stage of nanoindentation. Unloading of the indenter leaves a mixture of amorphous and metastable Si-III/Si-XII phases and a dislocation network in the plastically deformed zone of the Si crystal.

**Figure 18:**
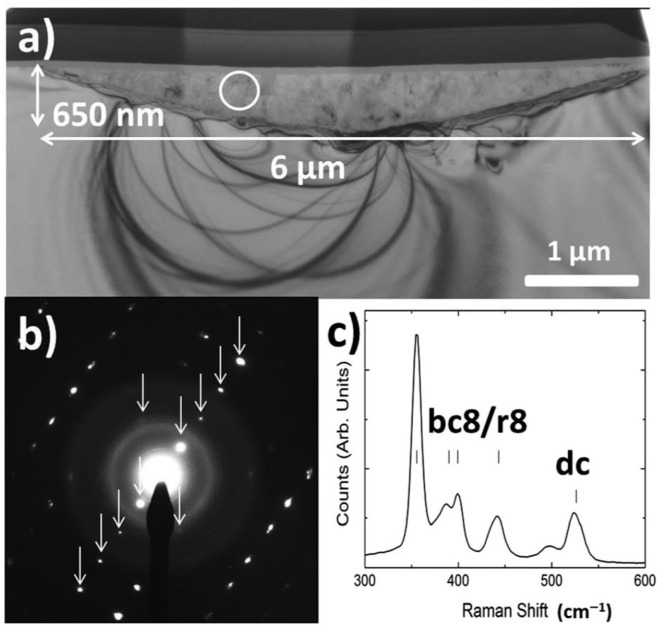
(**a**) A BF XTEM image of a residual indent formed with a 500 mN maximum load indentation using a ∼21.5 μm diameter spherical tip. (**b**) SADP taken from the circled region in (**a**) with arrows indicating diffraction spots arising from the bc8/r8 (Si-III/Si-XII) structure. (**c**) Raman spectrum of the residual indent impression confirming the presence of the bc8/r8 phases. Adapted with permission from Ref. [113].

The high-pressure silicon phases have attracted the attention of researchers. In particular, one called Si-XII. Indeed, DFT calculations have shown that the Si-XII phase is characterized by significantly higher electron mobility than in the semiconductor Si-I state [112], which may be important in the design of devices requiring materials with high charge carrier mobility. However, how can the stable SI-XII phase be introduced in the Si crystal structure? The answer to this question was provided by the works of Wong et al. [113,114,115,116]. They conducted experiments using a spherical diamond indenter (radius of 10.8μm). The phase composition of the permanently deformed crystal zone was then studied using transmission electron microscopy and Raman spectroscopy. As a result of applying the specialized indentation method, a dislocation-free region (6 μm wide and 650 nm deep, Figure 18) filled with a mixture of Si-III/Si-XII phases was obtained [113]. This discovery opens up possibilities for exploiting these phases for both optical and electrical applications. The work by Mannepalli et al. [117] demonstrated that modification of the solar cell absorbing layer with a grid of indents containing Si-XII phase resulted in an approximately 10 times improvement in the photocurrent density. The practical application of this interesting result will undoubtedly be the subject of future research, which should solve the problem of structural stability of a large network of micro-indents containing the Si-XII phase and, above all, the method of its production.

## Data Availability

No new data were created or analyzed in this study. Data sharing is not applicable to this article.
